# The use of photovoice in research with adolescents living with HIV in Africa: A scoping review

**DOI:** 10.4102/jphia.v16i1.625

**Published:** 2025-01-31

**Authors:** Yolanda R. Mayman, Brian van Wyk

**Affiliations:** 1 School of Public Health, Faculty of Community and Health Sciences, University of the Western Cape, Cape Town, South Africa

**Keywords:** adolescents, HIV, photovoice, participatory research, advocacy, Africa

## Abstract

**Background:**

Research involving adolescents living with HIV (ALHIV) is challenging, as adolescents often struggle with articulating their experiences, a difficulty further compounded by HIV-related stigma, particularly in African contexts. Photovoice methods offer a valuable participatory approach, engaging and allowing participants to express and share their stories through visual representation, amplifying their voices in research.

**Aim:**

The aim of this review is to map out and synthesise evidence on the use of photovoice methods in research with ALHIV in African contexts.

**Setting:**

All countries in the African region were included.

**Method:**

Eight electronic databases (ERIC, Ebscohost, PubMed, SCOPUS, CINAHAL, PsycINFO, CABI Direct and Africa Index Medicus) were searched to identify articles that used photovoice methods with ALHIV in Africa to publish between 2000 and 2024. The Preferred Reporting Items for Systematic Reviews and Meta-analyses (PRISMA) flowchart guided the screening and reporting of articles, with a narrative synthesis conducted.

**Results:**

Three key themes emerged in this review: resilience, personal challenges and environmental factors faced by ALHIV. Despite its strengths as a participatory approach, photovoice methods remain underutilised in research involving ALHIV in African contexts.

**Conclusion:**

This review demonstrates that high-quality photovoice studies can be effectively implemented in research with ALHIV in resource-constrained African settings with high HIV prevalence and stigma. Photovoice offers valuable insights to inform interventions aimed at improving the treatment outcomes and mental well-being of ALHIV, enhancing the relevance of such initiatives in these contexts.

**Contribution:**

These findings can further inform policies and interventions aimed at the care, well-being and treatment outcomes of ALHIV within African countries.

## Introduction

Adolescents represent the largest and fastest-growing population group globally, with a particularly rapid increase in African countries.^1^ The progression of the global human immunodeficiency virus (HIV)/acquired immunodeficiency syndrome (AIDS) pandemic has significantly contributed to the growing burden of HIV infection among adolescents aged 10–19 years, particularly in low- and middle-income countries.^2^ Adolescents living with HIV (ALHIV) face a unique set of challenges as they navigate living with a chronic illness while undergoing both physiological and psychological development.^3^ Additionally, adolescents and young people are more vulnerable to experiencing poor health outcomes in every phase of the HIV care continuum: testing, diagnosis, medication adherence and viral suppression.^4,5,6^ In 2022, an estimated 210 000 new HIV infections occurred among adolescents and young adults aged 15–24 years in Africa, where 82% of ALHIV reside, representing 88% of AIDS-related child deaths.^7,8^ Despite advancements in antiretroviral therapy (ART) that have transformed HIV into a manageable chronic disease, ALHIV continue to be affected by various illness-related stressors, including feelings of isolation, challenges in disclosing their HIV status and other emotional and behavioural difficulties.^9,10^ These stressors, both internal and external, are closely linked to mental health concerns. Internal stressors, such as shame and isolation related to their health or HIV status disclosure, can exacerbate fears of social rejection. External stressors, including social stigma and discrimination, often lead to bullying by peers, which negatively impacts treatment adherence.^11^

Community-based participatory research (CBPR) encompasses research methods that prioritise the inclusion of specific populations, such as people living with HIV (PLHIV), and value their subjective experiences.^12^ Creative research methods are often employed to gain deeper insights into the lived realities of vulnerable groups. One such method is photovoice, a participatory approach that integrates photography with qualitative research techniques.^13^ Photovoice engages community members in research by allowing them to express their lived experiences through photographs, fostering dialogue and promoting social change.^14,15,16^ Additionally, photovoice facilitates the sharing of diverse perspectives and lived realities, enhancing the understanding of individual experiences within vulnerable populations, such as children, in research.^17^

Photovoice methods have been widely used in health research to engage participants in reflecting on their subjective experiences.^17^ The core goals of photovoice include enabling individuals to document and reflect on their strengths, highlight community concerns, foster dialogue and advocate for policy changes that promote action.^18^ Health researchers have found photovoice to be particularly valuable in exploring sensitive topics, such as sexual and reproductive health, while also encouraging participant action and advocacy.^19^ The photovoice process involves using photography to capture the personal and collective experiences of marginalised individuals, providing them with an opportunity to convey their personal and community strengths and challenges. Participants engage in critical dialogue about the photos and share their perspectives through photo exhibitions to raise awareness and spark community change.^14,20,21^ Through this process, participants share and discuss images to express, advocate and amplify their experiences and ideas.^15^

The use of photovoice in public health research has grown significantly in recent years. Research findings demonstrate that photovoice methodology can be applied across various areas, including studies with individuals with physical disabilities, those living with HIV and those experiencing occupational performance challenges.^22,23^ Other reviews highlight how photovoice enables participants to share diverse perspectives and lived realities, fostering a deeper understanding of the individual experiences of vulnerable groups, such as children, in research.^17^

Historically, African contexts have been underrepresented in health research, with studies often being conducted ‘on’ rather than ‘with’ communities, resulting in limited direct benefits for the participants.^24^ The photovoice method presents a valuable alternative, as its cultural adaptability enhances participant engagement, programme effectiveness and long-term sustainability.^25^ Photovoice has been widely employed across various African countries to explore a range of health-related issues, including HIV, masculinity, resilience, mental health, safety and poverty. However, no review has yet synthesised the scope, objectives and impact of these studies. The purpose of this review is to consolidate the application of photovoice methods in research involving ALHIV within African contexts.

## Research methods and design

### Study design

This scoping review used the five steps as described by Arksey and O’Malley^26^ and Mak and Thomas.^27^ These five steps include: (1) identifying the review question, (2) identifying relevant studies, (3) selecting studies, (4) charting data and (5) summarising and reporting findings.

### Search procedure

A search strategy was created with the assistance of a health sciences librarian. A comprehensive database search was conducted in eight databases: Ebscohost, PubMed, SCOPUS, CINAHL, ERIC, PsycINFO, CABI Direct and African Index Medicus. Full-text articles were obtained using search strings containing key words using the ‘AND’ and ‘OR’ Boolean operators where appropriate. The search terms include: HIV, AIDS, adolescent, adolescence, teenagers, child, photovoice, photography, auto photography, photo-elicitation and visual participatory research method, health services and health services research. Title, abstract and full-text screening was conducted by both the authors for all articles included in this review.

### Inclusion and exclusion criteria

The PCC (Patient population, Concept and Context) framework, as recommended by the Joanna Briggs Institute (JBI), serves as a valuable guide for formulating clear and meaningful objectives and eligibility criteria for a scoping review.^28^ The inclusion criteria were based on this framework, where P = adolescents living with HIV; C = the use of photovoice methods; and C = an African setting or country. Research articles included in the review had to focus on adolescents aged 10–19 years living with HIV as the primary study population. Only original articles published in English in research journals between 2000 and 2024 were considered, ensuring the inclusion of relevant and up-to-date studies.

### Screening and study selection

**FIGURE 1:**
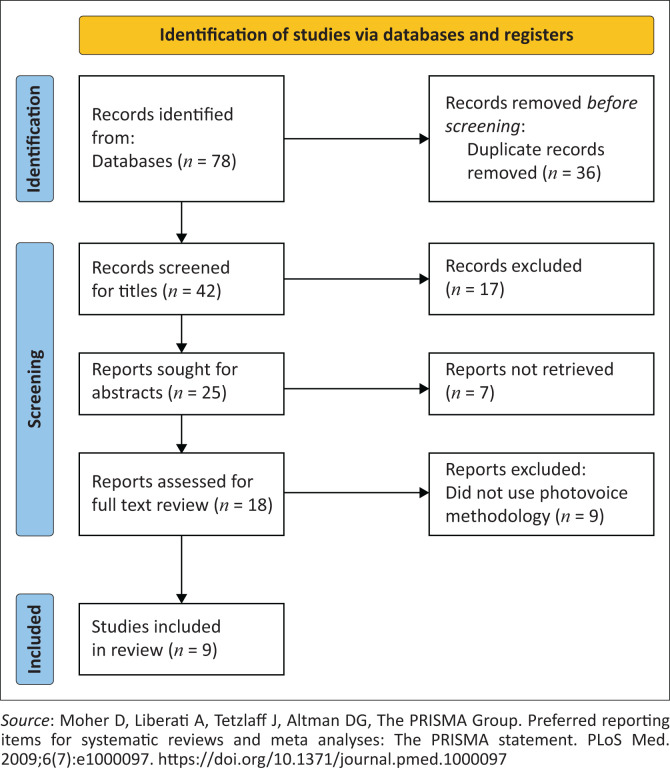
Preferred Reporting Items for Systematic Reviews and Meta-Analyses flow diagram for studies selection.

The inclusion criteria and search strategy were applied during the database searches. Both authors independently screened the titles and abstracts for eligibility. They also conducted full-text screening of the eligible studies, with no discrepancies identified. The selection process is illustrated in the Preferred Reporting Items for Systematic Reviews and Meta-analyses (PRISMA) diagram (see Figure 1)^29^.

### Data extraction

Both authors independently extracted data from the articles, organising the information on each article’s characteristics using a data extraction form. The form was developed collaboratively by both reviewers, who then cross-checked the included data items to ensure alignment with the review’s objectives. The data items extracted included the first author, year of publication, study location, whether the study was part of a larger research project, sample size, participant descriptions, health focus, key findings, photovoice elements, ethical considerations, trustworthiness and aspects of action or advocacy. The results of the data extraction are presented in Table 1 and Table 2, which detail the characteristics of the articles included in this review.

**TABLE 1:** Characteristics of included studies.

Source	Country	Part of alarger study	Sample size	Description of participants	Health focus	Study objectives
Adegoke andSteyn, 2017^36^	Nigeria	WAVE	5	Female participants had to show signs of resilience by attending HIV counselling and treatment centres, demonstrated by academic success or engagement in purposeful living, show evidence of social and emotional functioning and self-describe as resilient	Resilience	To explore how participants have been able to mitigate the negative effects of their experiences of HIV infection and experience resilience
Fournieret al., 2014^33^	Uganda	No	13	Children between the ages of 12 and 18 years living at the group home (*n* = 5 girls; *n* = 8 boys)	ALHIV	To explore the experiences of orphaned, HIV sero-positive children who live in a group home
Lovedayet al., 2022^30^	South Africa	No	14	ALHIV aged 16–19 years old with sub-optimal retention (defined as not taking ART for more than 30 days in the last 2 months)	Adherence	To describe the treatment journeys of ALHIV, and report on the enablers and barriers to on-going engagement with adherence and HIV services
Kimeraet al., 2020^34^	Uganda	No	11	12–19-year-old YLWHA with regular attendance to peer support group	HIV stigma	To explore lived experiences and effects of HIV-related stigma
Mukumbang andVan Wyk, 2020^16^	South Africa	Yes	21	ALHIV aged 10–19 years who are registered on ART at a public primary health care hospital	Adherence	To critically analyse mechanisms that hinder and facilitate adherence and engagement in HIV treatment
Orth andVan Wyk, 2021^31^	South Africa	Yes	18	Adolescents aged 10–19 years receiving ART at a public primary health care clinic. Individual interviews were also conducted with 5 health care workers	Health systems	To describe the implementation and experiences of a facility-based family support intervention (family clinic) in a public primary health care facility
Orth andVan Wyk, 2022^32^	South Africa	Yes	43	Participants living with HIV aged 15–19 years, receiving ART at one of 3 public primary health care facilities,	Mental wellness	To describe how ALHIV experienced taking ART talk about and understand mental wellness
Van Wykand Teti, 2020^15^	South Africa	Yes	10	Three groups: Group 1: 10–14 years, girls; Group 2: 15–19 years, girls; Group 3: 15–19 years, boys). Enrolled in ART at two urban public primary health care centres	Living with HIV and Adherence	(1) To explore experiences of living with HIV (2) To describe facilitators and barriers to ART
Vindevogel andKimera, 2023^35^	Uganda	Yes	11	Young people aged 10–24 years, being aware of their HIV status, having been on therapy for not less than 6 months, willing to participate in multiple sessions of group and individual discussions, and being able to speak either English or the local languages Lutooro or Luganda.	Social ecological resources for and resilience of youth living with HIV	To explore youths’ perceptions and representations of what promotes resilience

Note: Please see the full reference list of the article, Mayman YR, Van Wyk B. The use of photovoice methods in research with adolescents living with human immunodeficiency virus in Africa: A scoping review. J Public Health Africa. 2025;16(1), a625. https://doi.org/10.4102/jphia.v16i1.625, for more information.

HIV, human immunodeficiency virus; ART, antiretroviral therapy; ALHIV, adolescents living with HIV; YLWHA, youth living with HIV/AIDS.

**TABLE 2:** Characteristics of included studies.

Source	Photovoice training and instruction	Photovoice data collection	Ethical considerations	Other research methods	Main themes
Adegoke andSteyn, 2017^36^	Researchers re-examined an existing narrative data set from a qualitative multiple case study (WAVE) which includes a photo voice component with narratives	Not described	Not described	N/A	Resilience was characterised by framing positive, social competence and coping skills Challenges included: poverty, disrupted education, fear of disclosure of HIV status
Fournieret al., 2014^33^	Sessions: Safe and ethical use of cameras. A worksheet was provided to children to help them think about and write down strengths about themselves, their family, home and community was discussed	Group discussion SHOWeD framework Unspecified number of photos for group discussion	Parent/guardian consent Safe and ethical use of cameras	Focus groupdiscussions	Protective factors included: a sense of belonging appreciation for nature, home resources, community Hardships included stigma, psychological, emotional and social challenges
Lovedayet al., 2022^30^	Sessions: Discussed stigma, photovoice procedure, ethics of photography Participants were asked to take photos to portray their experiences of HIV-stigma and the effect of this stigma in their community for a period of 1 week	Group discussion	Ethical clearance was obtained Informed written consent parents/caretakers and a photo release form was signed by participants Pseudonyms were used throughout the audio recordings, analysis, reporting of results and all public exhibitions	SHOWeDquestioning guide	HIV-related stigma was experienced as being devalued, experiencing fear, experiencing injustices, feeling lonely and lacking future perspectives
Kimeraet al., 2020^34^	Two training workshops with seven participants in each were facilitated by two authors At the training workshops, which lasted 1.5 h, the background and rationale of the study were described, and each participant was provided with a cell phone with which to take pictures, and painting and/drawing materials. The idea of capturing experiences in an appropriate manner using either a cell phone or drawing or painting was introduced The week after the training course, participants captured images that symbolised their experiences of living with HIV in their community, together with something(s) that symbolised the challenges encountered and support they received during their treatment journey that impacted their adherence. After a week, they attended a Focus Group Discussion (FGD) led by two female authors at which they presented and discussed their pictures in more depth The FGDs were conducted in the local language (isiZulu) and lasted between 2.5 and 3 h	Focus group discussion	Ethical clearance was obtained. A waiver of parental consent was requested for only for participants older than 16 years	None	The data revealed four social domains which impacted adolescent adherence to ART: Social domain 1: the household; Social domain 2: the school; Social domain 3: the larger community; Social domain 4: the clinic
Mukumbang andVan Wyk, 2020^16^	The research team received 3-day training in photovoice method	Focus group discussion	Informed consent Consent from parents/caretakers for participants <18 years	None	Medication adherence behaviours included motivation, perceived support, sense of purpose and spiritual activities Medication non-adherence behaviours included perceived stigma, negative peer pressure, mistrust and unbelief, frustration and depressive feelings
Orth andVan Wyk, 2021^31^	In the first session, participants received consent forms and a cell phone. The research team provided additional information regarding the study and utilisation of the phones. Participants were then instructed to take at least five pictures that tell their story of their experiences with ART	Group discussion	Institutional research board ethical approval was obtained Informed consent sheets were obtained Adolescents younger than 18 were required to obtain consent from their parent/guardian	Individual interviews with health workers	Health systems interventions: ‘Family Club’, ‘Risk of Treatment Failure’ (ROTF) clinic. HIV-related challenges included disclosure of HIV status, motivation to adhere to treatment, challenges with medication adherence, family support and disclosure of HIV status to friends
Orth andVan Wyk, 2022^32^	The research team received training in photovoice techniques and procedures. Three contact sessions were made with adolescent participants. In the first contact session, the health worker gatekeeper introduced the researchers to eligible adolescent participants and the researchers described the research to the participants and invited their participation During the second meeting, adolescents returned with their consent forms and were provided with cell phones with camera capabilities At the final meeting date, the group met and presented their photos, followed by a discussion on their photos	Group discussion	Informed consent and assent was obtained from parents/guardian and child/adolescent	None	Mental wellness concepts: connectedness, spirituality and mindfulness, social coherence and awareness, self-esteem, self-acceptance, sense of coherence Behaviours indicating mental wellness: self-efficacy, coping, resilience, life purpose, engagement in enjoyable activities and physical functioning
Van Wykand Teti, 2020^15^	Study staff instructed participants to tell their story of taking HIV medicines, focusing on the strengths and challenges of taking medicine, with photos. They gave each participant a camera, taught participants to use the camera, and led a discussion to make sure participants had a plan to move forward	Group discussion Unspecified number of photos and meanings for discussion	Institutional research board ethics approval	None	Themes included motivations for taking medicine, forming an identity with HIV, managing disclosure and treatment, relationships between living conditions and HIV treatment Treatment motivation was informed by familial support participants received
Vindevogel andKimera, 2023^35^	The first session aimed to establish rapport and introduce participants to the concept and procedure of photovoice. During this session, participants were also acquainted with representing themselves through photographs The second session focussed on resilience and guided participants to adopt a resilience lens to identify and appreciate the resources that strengthen them In the third session, the participants conceived and visualised images representing the resilience-enabling resources in the form of hand-drawn pictures as precursors for the photographs. At the end of this session, each participant was given a digital camera to take photographs documenting resilience in their everyday lives. They carried the camera with them and had the opportunity to take photographs until the next session. Accompanying narratives were either written down in a notebook or recorded audially by the participants In the fourth and fifth sessions, batches of photographs were received from participants. The photos were extracted from the cameras into a computer by the researchers and stored in different folders clearly marked with each participant’s pseudonym. Participants were then asked privately to select photos they wanted to present in a group discussion and those they wanted to present to researchers only. All participants choose to present their selected photos in a group discussion. In the sixth and seventh sessions, participants discussed their selected photos	Group discussion using a variation of the ‘SHOWeD’ technique for facilitation of photovoice discussions	Ethical clearance was obtained. Written informed consent to participate in this study was provided by the participants’ legal guardian/next of kin for the publication of any potentially identifiable images or data included in this article	None	Participants experience well-being amidst HIV-related adversity through managing tensions in material resources, a sense of identity, power and control in their lives, cultural adherence, relationships, a sense of cohesion and social justice

Note: Please see the full reference list of the article, Mayman YR, Van Wyk B. The use of photovoice methods in research with adolescents living with human immunodeficiency virus in Africa: A scoping review. J Public Health Africa. 2025;16(1), a625. https://doi.org/10.4102/jphia.v16i1.625, for more information.

HIV, human immunodeficiency virus; ART, antiretroviral therapy; N/A, not appliable.

### Risk of bias and quality assessment

The quality of the articles was assessed using an adapted Critical Appraisal Skills Programme Qualitative Checklist (CASP Tool), which included the components of photovoice research (see Table 3). Analysis focussed on the quality of the articles as well as how they met the core tenets of photovoice methods. The checklist assisted in the classification and evaluation of the articles in terms of the research question, photovoice method, the data collection processes, ethical issues, research value and action. The response options for the CASP tool and photovoice tool were scored as: yes (1), no (0) and can’t tell (0.5).

**TABLE 3:** Critical appraisal skills programme qualitative checklist (CASP Tool) and photovoice tool checklist.

CASP tool	Source								
	Adegoke andSteyn, 2017^36^	Fournieret al., 2014^33^	Kimeraet al., 2020^34^	Lovedayet al., 2022^30^	Mukumbang and Van Wyk, 2020^16^	Orth andVan Wyk, 2021^31^	Orth andVan Wyk, 2022^32^	Van Wykand Teti, 2020^15^	Vindevogel and Kimera, 2023^35^
Clear statement of research aims	0	0	0	0	1	1	1	0	1
Photovoice method appropriate; photos justified	1	1	1	1	1	1	1	1	1
Photovoice used to answer research question	1	1	1	1	1	1	1	1	1
Recruitment strategies appropriate for aims	1	1	1	1	1	1	1	1	1
Data collected to address research issue	1	1	1	1	1	1	1	1	1
Relationships between researcher and participants were considered	0	0	1	0	1	1	0	0	0
Ethical issues taken into consideration	1	1	1	1	1	1	1	1	1
Rigorous analysis	1	1	1	1	1	1	1	1	1
Clear statement of findings	1	1	1	1	1	1	1	1	1
Valuable research findings	1	1	1	1	1	1	1	1	1
Photovoice tool facilitated a process for participants to record and reflect experiences	0	1	1	1	1	1	1	1	1
Promoted critical dialogue through group discussion of photos	1	1	1	1	1	1	1	1	1
Resulted in action or advocacy	0	1	0	0	0	1	1	1	0
Evidence that findings could be/were applied to practice	0	1	1	1	1	1	1	1	1

**Total**	**9**	**10**	**11**	**10**	**12**	**12**	**11**	**10**	**11**

Note: Please see the full reference list of the article, Mayman YR, Van Wyk B. The use of photovoice methods in research with adolescents living with human immunodeficiency virus in Africa: A scoping review. J Public Health Africa. 2025;16(1), a625. https://doi.org/10.4102/jphia.v16i1.625, for more information.

### Data synthesis

The results of this review were synthesised narratively. The authors summarised and synthesised the characteristics and photovoice description of each of the included studies in Table 1 and Table 2.

### Ethical considerations

Ethical approval was obtained from Biomedical Science Research Ethics Committee (reference number: BM22/7/4). This forms part of a larger project. Participant consent was not obtained because this study had no participants.

## Results

### Characteristics of included studies

The screening and selection process is illustrated in Figure 1. Database searches yielded a total of 78 results. A total of 36 duplicate articles were removed and 42 articles remained for consideration. After the title and abstract screening 24 were excluded (because of not meeting the full inclusion criteria) and the remaining 18 articles were eligible for full-text screening. Both authors read the full text articles and met to discuss each full text article considered for inclusion. Subsequently, nine articles were further excluded because of not using photovoice methods and a final nine articles were retained for analysis.

The majority of studies (*n* = 5) were conducted in South Africa,^15,16,30,31,32^ followed by three studies in Uganda^33,34,35^ and one in Nigeria.^36^ These studies were published between 2014 and 2023. Most studies (*n* = 7) recruited adolescent participants from healthcare facilities,^16,30,31,32,34,35^ while one study gathered participants from a group home,^33^ and another utilised an existing narrative dataset from a qualitative case study involving adolescent girls living with HIV.^36^

The sample sizes in the articles varied from small to medium to large, as classified by Marshall et al.^37^ In qualitative research, a sample size of fewer than 20 participants is considered small, a sample of 20–40 participants is categorised as medium to large, and a sample of 41–50 participants is regarded as large. Sample sizes of 20–30 participants are typically used in qualitative research to achieve data saturation.^38^ Based on this classification, eight articles had small to medium sample sizes, ranging from 5 to 21 participants,^30,31,33,34,35,36^ while one article analysed a larger sample of 43 participants.^32^ Eight of the nine studies included both adolescent girls and boys,^15,16,30,31,33,34,35^ while one study focussed exclusively on girls.^36^ Notably, none of the included studies conducted gender-sensitive analyses.

All of the included articles utilised photovoice methods to collect data and explore the subjective experiences of ALHIV across various African settings. Two studies examined instances of resilience among ALHIV,^35,36^ while another focussed on the experiences of orphaned sero-positive children.^33^ One study investigated the impact of HIV stigma on the lived experiences of ALHIV,^34^ and three studies addressed the various factors and barriers affecting adherence to and engagement in HIV treatment among ALHIV.^15,16,30^ One study explored the role of health systems and the experiences of a family support intervention,^31^ while another focussed on the mental wellness of ALHIV.^32^

### Themes of included studies

Three overarching themes emerged from the articles included in this review: resilience, personal challenges and environmental factors (Table 2). Four articles highlighted resilience among ALHIV, demonstrating how it served as a protective factor, helping to mitigate adversity and enhance both their well-being and treatment outcomes.^15,33,35,36^

Five articles discussed the personal challenges faced by ALHIV.^15,30,33,34,36^ These challenges encompassed fear of disclosing HIV status, struggles with identity as individuals living with HIV, HIV-related stigma, self-awareness and personal growth, and emotional difficulties such as fear and anger. Additional challenges included a lack of future prospects, loneliness, poverty, parental unemployment, teenage pregnancy and experiences of injustice in various settings, including the workplace, home and school. The studies indicated that the dynamics of relationships within the household and social environments, along with the knowledge internalised by ALHIV, significantly influence their behaviour and treatment adherence. A lack of support and open discussion about HIV and ART within the household served as significant barriers to retention in care, as family members were often unaware of the adolescent’s HIV status. Furthermore, financial constraints within the household adversely impacted ALHIV’s adherence to treatment and their access to necessary care.

The larger community plays a crucial role in either promoting or hindering treatment adherence among ALHIV. Two articles highlighted environmental factors that influenced treatment adherence.^15,30^ Factors such as access to clean water, nutritious food, adequate living conditions, transportation, relationships, healthcare facilities, healthcare providers, and access to information about HIV and ART were identified as directly impacting the health outcomes of ALHIV. Relationships within the community and healthcare settings either supported or impeded adherence to treatment. Positive support and encouragement from these relationships were found to significantly enhance ALHIV’s commitment to treatment and retention in care.^30^

### Summary of photovoice components of included studies

The goals and application of the photovoice methodology were highly appropriate for the articles in this review, as it facilitated the documentation of the challenges faced by ALHIV and contributed to the development of solutions to address these challenges. In one study, participants were asked to take photographs that reflected their adjustment to living with HIV.^36^ In four other studies, participants were provided with digital cameras or phones to capture images depicting their experiences with HIV-related stigma, the challenges they faced and the resilience they demonstrated as ALHIV.^30,33,34,35^ The remaining four studies equipped participants with cell phones featuring high-resolution cameras to document their experiences of living with HIV and undergoing ART.^15,16,31,32^

Eight studies included in this review incorporated introductory sessions during which the study and its objectives were explained to participants. Four of these studies conducted sessions where the study was summarised and consent procedures were reviewed during the first meeting.^15,31,32,34^ In addition, three studies implemented photovoice process sessions, which involved training on the ethical and safe use of cameras, as well as guidance on developing themes for the photographs.^30,33,35^ One study, in contrast, employed inductive analysis of photovoice data derived from an existing narrative dataset.^36^

The number of photographs participants were asked to share varied across studies. Three studies indicated that participants selected five photographs they wished to discuss and share with researchers and peers,^31,32,34^ while another study reported that an average of five photographs were collected from each participant.^16^ One study specifically documented the number of photos presented by each participant.^36^ The remaining studies did not specify the quantity of photographs shared.^15,30,33,35^ Participants shared and discussed their photographs either in group discussions^16,33,34,35^ or individual interviews.^36^

### Assessment of the photovoice components of the included studies

Eight studies allowed study participants to record and reflect on their experiences. All included studies also promoted critical dialogue by having traditional group discussions where participants were able to share their photographs. One study included additional qualitative individuals interviews with healthcare workers.^31^ Action or advocacy was evident in three studies through photo exhibitions and the recommendation that photovoice research be utilised as a valuable tool for understanding HIV medication adherence, as well as a potential supportive intervention warranting further exploration.^15,32,33^ Additionally, eight studies reported how their findings could be, or were, applied to practice.^15,16,30,31,32,33,34,35^

### Appraisal of the quality of the included studies

The overall scores obtained from applying the CASP tool to the included articles ranged from 8 to 10 out of 10, signifying that the studies were of high quality. The use of the photovoice method, including the application of photographs, was deemed both appropriate and well-justified for addressing the research questions in all studies. Recruitment strategies and data collection methods were found to be suitable for achieving the research objectives and addressing the core issues. However, only two studies explicitly discussed the relationship between researchers and participants,^16,34^ while the others did not report on this aspect. All studies thoroughly addressed ethical considerations, including informed consent, institutional ethical approval and the ethical management of photographs. The analysis in each study was rigorous, and all presented a clear statement of findings. Based on these factors, it can be concluded that all studies contributed valuable insights into the use of photovoice methods for documenting and reporting the experiences of ALHIV in African contexts.

## Discussion and limitations

The review of photovoice studies is particularly significant, as photovoice methods have been utilised in health research to highlight issues related to health equity and to address the social determinants of health.^39^ A scoping review demonstrated that photovoice is an effective tool for exploring the experiences of individuals at risk.^23^ Additionally, a study conducted in Namibia on the experiences of individuals with disabilities found that photovoice provided a platform for these individuals to voice their concerns and engage with policymakers.^40^ Other findings suggest that ethical considerations regarding populations with illnesses and disabilities require further scrutiny.^23^ This is evident in the articles included in this review, as all reported on ethical issues related to the inclusion of vulnerable groups as participants.

Findings showed that ALHIV may rely on protective factors, such as a sense of belonging, appreciation for nature, resources and community, which increase their resilience and help mitigate the challenges they may experience. As young people transition into adolescence and adulthood, they encounter a range of complex challenges, including adherence to ART, disclosure of their HIV status and the stigma they may anticipate or experience.^41^ One study in this review found that photovoice is particularly effective for facilitating dialogue on sensitive topics with youth, such as living with HIV.^42^ A systematic review in 2020 highlighted that photovoice provides a platform for representing the voices of individuals within communities who are often marginalised or unheard.^43^ The role of family and household dynamics in shaping the experiences of ALHIV was particularly significant. Studies revealed that a lack of support and open communication about HIV and ART within households was a key barrier to retention in care, with many adolescents’ HIV status remaining undisclosed to family members. Furthermore, financial constraints within the household negatively affected access to treatment and adherence to care, echoing the findings of studies that emphasise the intersection of socioeconomic challenges and health outcomes for ALHIV. These findings highlight how environmental conditions and access to essential resources have a direct impact on the well-being of ALHIV as research indicates that contexts of poverty can significantly undermine both the educational and psychosocial well-being of ALHIV.^44^ Consequently, the development of strategies and interventions aimed at enhancing the well-being of ALHIV should be prioritised by both local and international agencies and organisations. These findings suggest that photovoice methods are valuable tools for amplifying the voices of ALHIV, enabling them to share their experiences.

Photovoice is recognised for its flexibility in capturing and highlighting the perspectives of vulnerable individuals.^19^ Given this adaptability, it is crucial that the processes and methods employed are clearly defined to ensure participants can express themselves fully and safely, ultimately benefiting from the research. Ethical considerations in photovoice research include issues related to consent, data anonymity, participant confidentiality and the use and distribution of visual data.^45^ These aspects must be carefully addressed to uphold best practices and ensure positive research outcomes for populations such as ALHIV. Additionally, the relationship between the researcher and the participant should be explicitly outlined in photovoice studies. This element of CBPR is essential for validating participant voices, fostering meaningful dialogue, promoting the exchange of information and ensuring reflexivity.^12,23^

This review is not without limitations. The number of articles included is relatively small, primarily because of the study’s focus on the use of photovoice methods in research with ALHIV in Africa. Both the specific inclusion criteria and the limited research in this area further constrained the number of studies considered. While including more articles could have potentially strengthened the findings, the results of this review warrant the need for further research using photovoice methods with ALHIV. This methodology has proven to be an effective tool for such studies, underscoring the importance of continued exploration in this area.

## Conclusion

This review demonstrates that photovoice methods are a valuable tool for exploring the experiences, perspectives and challenges faced by ALHIV across the African continent. The findings underscore the ability of photovoice to address various factors influencing treatment adherence and the overall mental well-being of ALHIV. Given its capacity to amplify the voices of vulnerable populations such as ALHIV, it is strongly recommended that future research continue to utilise photovoice. This approach not only deepens our understanding of ALHIV’s experiences but also facilitates the integration of their perspectives into research, policy and interventions. Such efforts can ultimately enhance our knowledge of the impact of HIV on treatment outcomes and mental health, leading to more effective, youth-centred strategies for supporting ALHIV.
